# A nociresponsive specific area of human somatosensory cortex within BA3a: BA3c?

**DOI:** 10.1016/j.neuroimage.2020.117187

**Published:** 2020-11-01

**Authors:** Rosa M. Sanchez Panchuelo, Sally Eldeghaidy, Andrew Marshall, Francis McGlone, Susan T. Francis, Oleg Favorov

**Affiliations:** a Sir Peter Mansfield Imaging Centre, School of Physics and Astronomy, University of Nottingham, Nottingham NG7 2RD, UK; b Future Food Beacon, School of Biosciences, University of Nottingham, Nottingham, UK; c Institute of Aging and Chronic Disease, University of Liverpool, Liverpool, UK; d School of natural Science and Psychology, Liverpool John Moores University, Liverpool, UK; e NIHR Nottingham Biomedical Research Centre, University of Nottingham, Nottingham, UK; f Department of Biomedical Engineering, University of North Carolina, CB #7575, Chapel Hill, NC 27599, USA

**Keywords:** Somatosensory cortex, Area 3a, Slow second pain, fMRI, Thermonoxious stimulation

## Abstract

It is well recognized that in primates, including humans, noxious body stimulation evokes a neural response in the posterior bank of the central sulcus, in Brodmann cytoarchitectonic subdivisions 3b and 1 of the primary somatosensory cortex. This response is associated with the 1st/sharp pain and contributes to sensory discriminative aspects of pain perception and spatial localization of the noxious stimulus. However, neurophysiological studies in New World monkeys predict that in humans noxious stimulation also evokes a separate neural response—mediated by C-afferent drive and associated with the 2nd/burning pain—in the depth of the central sulcus in Brodmann area 3a (BA3a) at the transition between the somatosensory and motor cortices. To evoke such a response, it is necessary to use multi-second duration noxious stimulation, rather than brief laser pulses. Given the limited human pain-imaging literature on cortical responses induced by C-nociceptive input specifically within BA3a, here we used high spatial resolution 7T fMRI to study the response to thermonoxious skin stimulation. We observed the predicted response of BA3a in the depth of the central sulcus in five human volunteers. Review of the available evidence suggests that the nociresponsive region in the depth of the central sulcus is a structurally and functionally distinct cortical area that should not be confused with proprioceptive BA3a. It is most likely engaged in interoception and control of the autonomic nervous system, and contributes to the sympathetic response to noxious stimulation, arguably the most intolerable aspect of pain experience. Ablation of this region has been shown to reduce pain sensibility and might offer an effective means of ameliorating some pathological pain conditions.

## Highlights

• First 7T fMRI study of C-afferent nociceptive input in human S1.
• Thermonoxious BOLD responses found anterior to innocuous vibrotactile maps in S1.
• Nociresponsive region within area 3a in line with neurophysiological animal studies.

## 1 Introduction

In primates, noxious body stimulation evokes neural activity in a distributed network of cortical regions, including primary and secondary somatosensory areas, insula, anterior cingulate cortex (ACC), and prefrontal cortex, each region making its own contribution to discriminative, cognitive, or affective/motivational aspects of the evoked pain (Apkarian et al., 2005; Iannetti and Mouraux, 2010). The primary somatosensory cortex (S1) is widely recognized as making a sensory discriminative contribution to pain perception (Bushnell et al., 1999; Ploner et al., 1999; Schnitzler and Ploner, 2000).

S1 in humans and other primates is subdivided into four distinct cytoarchitectonic Brodmann areas (BA1, BA2, BA3a, BA3b), occupying the crown of the postcentral gyrus (BA1), the anterior bank of the postcentral sulcus (BA2), and the fundus (BA3a) and posterior bank (BA3b) of the central sulcus. Each area exhibits distinct functional properties, reflecting the diversity of somatosensory receptor submodalities innervating the body. Neurons with functional properties indicative of their involvement in discriminative representation of nociceptive stimuli are found in BA1 and BA3b (Chudler et al., 1990; Kenshalo et al., 2000; Ploner et al., 2000). These nociresponsive neurons are dominated by Aδ nociceptive afferent drive and respond with stimulus-response characteristics fully consistent with their mediation of the early, sharp, and well-localized percept designated as “fast/discriminative pain” (Kenshalo and Willis, 1991). These neurons do not form nociception-pure cortical columns, but are interspersed among other neurons that are innervated exclusively by Aβ mechanoreceptors and are responsible for the discriminative touch.

Discriminative pain processing is performed by S1 in a region that occupies the crown of the postcentral gyrus and upper levels of the posterior bank of the central sulcus. This, however, is not a full story. Studies performed in near-lissencephalic New World squirrel monkeys (Chen et al., 2009; Tommerdahl et al., 1996, 1998; Whitsel et al., 2009; Yang et al., 2018) have shown that another region of S1 is also engaged by nociceptive stimulation, but in a very different manner (Vierck et al., 2013; Whitsel et al., 2019). This region is located most anteriorly in S1, at its interface with the primary motor cortex (M1). Cytoarchitectonically, it occupies a transitional zone between S1 and M1, characterized by a blend of motor and sensory features (Jones and Porter, 1980). In primates, this transitional zone is usually assimilated with BA3a. Neurons in this anterior nociresponsive region respond most vigorously to C-nociceptor afferent drive, exhibit prominent slow temporal summation and prolonged after discharges in response to repetitive or continuous noxious skin heating stimulation, and are minimally affected by non-noxious tactile or proprioceptive stimuli (Whitsel et al., 2009). Behavior of these neurons is closely related to the slow, 2nd/burning pain mediated by C-nociceptors, rather than to the fast, 1st/discriminative pain mediated by Aδ nociceptive afferents. To distinguish between these two nociresponsive regions in S1, we will refer to them as the “fast Aδ-dominated nociresponsive region” in BA3b-BA1 and the “slow C-dominated nociresponsive region” at the S1-M1 border. Available evidence suggests that the slow C-dominated nociresponsive region plays an important role—different from that of the fast Aδ-dominated region—not only in normal nociception, but also in some chronic pain disorders (Vierck et al., 2013; Whitsel et al., 2019).

In humans, the transitional zone between S1 and M1 lies at the fundus of the central sulcus, although its precise location varies significantly among individuals (Geyer et al., 1999). So far, the inference from nonhuman primate studies that this region in humans might be responsive to noxious stimulation has received limited experimental attention, with only a few human studies exploring this possibility. Using a grid of subdural electrodes, Baumgartner et al. (2011) showed that noxious laser-evoked potentials were generated in BA3a. In contrast, Gelnar et al. (1999) found painful heat-evoked activation to be concentrated mainly in BA1 in their fMRI study and concluded that there was no evidence of activation of neurons in BA3a. Chen et al. (2002), on the other hand, found large inter-individual variability in the S1 location of fMRI responses to painful heat stimuli—some in the depth of the central sulcus, some on the postcentral crown. Finally, Yoo et al. (2007) reported that BA3a was selectively activated in their fMRI study with the use of acupuncture stimuli, which evoked a “dull achy sensation”.

Other than these studies, the human pain imaging literature has not explored the possibility of BA3a involvement in nociception. Some imaging studies delivered noxious stimuli by using single laser pulses (Bingel et al., 2003; Mancini et al., 2012; Ploner et al., 2000, 2002; Qiu et al., 2006), which are too brief to build substantial activity in the slow C-dominated nociresponsive S1 region. Other studies had low spatial resolution (Andersson et al., 1997; Kanda et al., 2000; Jin et al., 2018; Nahman-Averbuch et al., 2014). Yet other studies were not interested in discriminating among individual Brodmann areas within S1 or did not provide detailed information on the spatial extent of the noxious responses they evoked in the central sulcus (Becerra et al., 2001; Bingel et al., 2003; Downar et al., 2003; Fairhurst et al., 2012; Hu et al., 2015; Ibison and Vogt, 2013; Moulton et al., 2012; Ploner et al., 2002; Tseng et al., 2013; Upadhyay et al., 2010).

To address such limitations, here we use high spatial resolution (1.5 mm isotropic) fMRI at 7T and relatively long (5s duration) thermonoxious skin stimulation on human volunteers to test the prediction of a neural response in the depth of the central sulcus, in a neighborhood of the border between S1 and M1. The findings of this study are discussed through a comparative review of the association of this region with nociception in rodents and non-human primates, and its significance to normal and pathological pain.

## 2 Methods

Five subjects (2 females, age: 36±4 years (mean ± SEM)) participated in at least two fMRI sessions, one session to measure thermonoxious evoked BOLD responses to thermonoxious stimulation of the skin on either the palm's thenar eminence or the fingertips, and a second to generate mechanoreceptive somatotopic maps of the hand digits using vibrotactile stimulation delivered to the fingertips. Two subjects participated in additional thermonoxious fMRI sessions (see Table 1 for full details of scanning sessions). Scanning was performed on a 7T Achieva MR system (Philips Healthcare; Best, Netherlands) using a head volume transmit coil and a 32-channel receive coil (Nova Medical: Wilmington, MA). Experimental procedures for all studies were approved by the University of Nottingham Medical School's Ethics Committee. All subjects gave written informed consent. None of the subjects had a history of neurological disorders.

**Table 1:** Details of thermonoxious fMRI scan sessions. (*) Data acquired with the painless heat paradigm.

	Session	Data collected	Temperature	GSR collected inside 7T	Perception
Subject 1	1	Thenar: 2 fMRI runs	46 °C	Yes	Very Painful
		Digits: 2 fMRI run	47.5 °C	Yes	Very painful
	2	Thenar: 2 fMRI runs	46 °C	No	Very painful
		Digits: 1 fMRI run	48 °C	No	Very painful
	3	Thenar: 2 fMRI runs	46 °C	Yes	Very painful
		Digits: 3 trials	48.5 °C	No	Very painful
	4	Thenar: 2 fMRI runs	46.5 °C	No	Very painful
		*Thenar: 2 fMRI runs	42 °C	No	Hot, not painful
Subject 2	1	Thenar: 2 fMRI runs	45 °C	No	Very painful
		Digits: 2 fMRI runs	46.5 °C	No	Very painful
	2	Thenar: 2 fMRI runs	44 °C	No	Very painful
Subject 3	1	Thenar: 2 fMRI runs	43.5 °C	No	Very painful
		Digits: 2 fMRI runs	46 °C	No	Not very painful
Subject 4	1	Thenar: 2 fMRI runs	46.5 °C	No	Not as painful as digits
		Digits: 2 fMRI runs	47 °C	No	Very painful
Subject 5	1	Thenar: 2 fMRI runs	47 °C	No	Not as painful as digits
		Digits: 2 fMRI runs	48.5 °C	Yes	Very painful

### 2.1 Stimulation paradigm

**Fig. 1:**
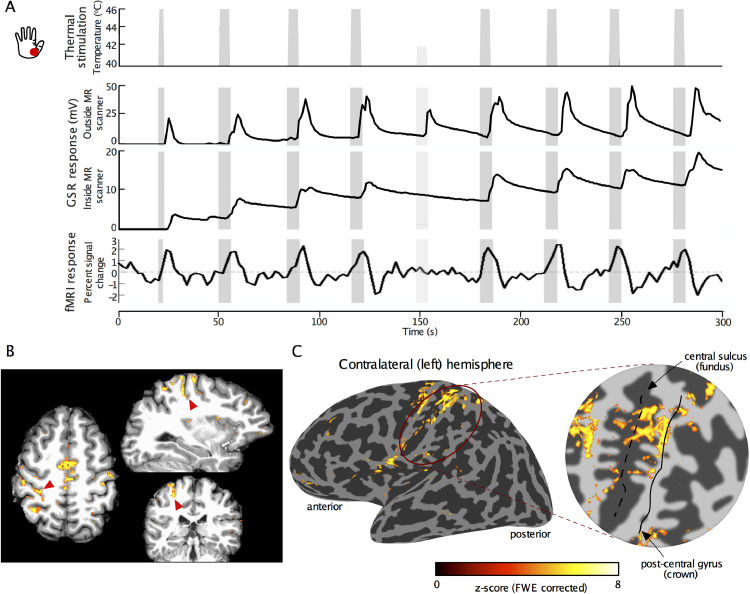
(A) Thermonoxious stimulation paradigm (top) and a corresponding exemplary GSR response collected outside and inside the 7T MR scanner, together with associated BOLD fMRI response time series in BA3a and BA3b of S1. The first thermonoxious trial in a run was of shorter duration (2.5 s) than the rest of trials (5s). Within each run there was a dummy trial (at 42 °C; indicated by the lighter grey bar), which did not evoke any painful sensation, as reflected by a lower response in the GSR trace. In contrast, painful stimulation (for this subject at 46 °C) evoked strong GSR responses. Notice that the dummy trial did not evoke any BOLD response. (B) Exemplary sections of fMRI activation pattern (*z*-score >3.08, FWE corrected) evoked by thermonoxious stimulation of the contralateral thenar eminence in Subject 1, overlaid on their T1-weighted structural scans. Red arrowheads point to the location of the fundus of central sulcus in anatomical space. (C) fMRI activation map (46 °C thenar stimulation, Subject 1) overlaid on the inflated cortical surface and zoomed representation of the central sulcus for the contralateral (left) hemisphere. Dark grey cortical regions represent sulci, whilst light grey represent gyri. The black dashed and solid lines in the flattened patch indicate the central sulcus fundus and the postcentral gyrus midline respectively.

Thermonoxious skin stimulation was delivered to the right hand using a CHEPS probe (Pathway, Medoc, RamatYishai, Israel) with a 27 mm diameter (572 mm^2^ contact area) MRI-compatible Peltier thermode. This probe was chosen as it offers fast heating rates of up to 70 °C/s within a temperature range of 30 °C to 55 °C, enabling the delivery of painful heat stimuli in less than 300 ms. Painful stimulation was applied to the skin of either the thenar eminence at the base of the thumb or the fingertips of digits 2 and 3. Painful stimulation was achieved by rapidly increasing the temperature from a baseline of 40 °C to a desired noxious level (up to 49 °C) for 5 s and then returning to a baseline temperature of 40 °C (see Fig. 1). The temperature of 40 °C was chosen to have a warm stimulus as the baseline (rather than neutral) in order to minimize non-noxious heat component of the stimulus and facilitate distinction of the effects of painful heat versus warm heat in BOLD responses.

Prior to each scanning session, each subject underwent an evaluation to determine the temperature to evoke significant but tolerable pain sensations at each stimulation site. For this, the stimulator's heating plate was placed in continuous contact with the skin, delivering a constant baseline temperature of 40 °C. Every 30 s, the plate temperature was raised and held for 5 s before returning to the baseline. Such thermal ramp stimuli started at a low temperature of 42 °C and increased by 0.5 °C at every consecutive trial, during which the subject provided a pain level rating on a scale from 0 (no pain) to 10 (maximally painful). Stimulation trials continued until a rating of 7 was reached. The temperature that produced this rating was then used in the scanning sessions. Galvanic skin responses (GSR) were collected throughout this thermal stimulation period to monitor the degree of the experienced pain, with the two electrodes placed on the non-stimulated (left) hand (GSR module, Brain Products GmbH, Gilching, Germany).

The fMRI paradigm was performed with the target temperature producing a pain rating of 7. The paradigm was a block design with 5 s periods of exposure to the target noxious temperature, interleaved with baseline periods at non-noxious 40 °C of varying inter-stimulus interval (Fig. 1.A). Each fMRI run comprised 8 trials of thermonoxious stimulation (with first trial lasting 2.5 s) and a dummy trial at 42 °C (non-painful thermal stimulation). Each fMRI run lasted 5 min and was repeated twice at each skin location (thenar eminence and fingertips). In order to compare the effects of thermonoxious stimulation with non-noxious thermal stimulation, one subject also underwent a non-painful heat paradigm using a baseline temperature of 37 °C and a target temperature of 42 °C, with the same stimulation timings as used for the thermonoxious paradigm. Two fMRI runs of this painless thermal stimulation, in which heating was applied to the thenar eminence, were acquired prior to two fMRI runs of thermonoxious stimulation applied to the same skin site.

For the mechanoreceptive responses, a ‘travelling wave’ paradigm comprising non-noxious vibrotactile stimulation was used to map the representation of the individual digits in the contralateral S1 (Besle et al., 2013; Sanchez-Panchuelo et al., 2010). Vibrotactile stimulation was delivered to a ~1 mm^2^ skin area on the distal phalanges (digit tips) of the right hand using five independently controlled piezoelectric devices (Dancer Design, St. Helens, UK). Each of the five digits were sequentially stimulated in either a forward (from digit 1 to digit 5) or backward (from digit 5 to digit 1) ordering for 8 cycles. Each vibrotactile stimulus lasted 4s and consisted of bursts of 0.4 s duration at 30 Hz stimulation frequency separated by 0.1 s gaps, resulting in a 20 s stimulation cycle.

### 2.2 Acquisition

Functional MRI data were acquired using a T_2_*-weighted, multi-slice, single-shot gradient echo–echo planar imaging (GE-EPI) multiband acquisition with 1.5 mm isotropic resolution and a field of view of 192 × 192 mm^2^ in the anterior-posterior and right-left directions (SENSE acceleration factor 1.5 in the anterior-posterior direction, Partial Fourier factor of 0.8, echo time TE=25 ms). A multiband factor of 3 was used to collect 54 slices in a TR of 2 s. Throughout, respiratory and cardiac responses were monitored using a pneumatic belt placed around the upper abdomen and a Peripheral Pulse Unit (PPU) on the left index finger. fMRI runs were followed by the acquisition of (i) two spin-echo EPI reference scans (each 3 volumes, with acquisition matrix, echo spacing and bandwidth matched to fMRI acquisition), one with matched and one with reversed phase-encoding direction, for subsequent distortion correction using TOPUP within FSL (http://fsl.fmrib.ox.ac.uk/fsl/fslwiki/topup), (ii) a high-resolution T_2_*-weighted axial FLASH dataset with the same slice prescription and coverage as the functional data (0.5 × 0.5 mm^2^ in-plane resolution; TE/TR = 9.3/458 ms, FA = 32°, SENSE factor = 2). This facilitated subsequent registration of the fMRI data to a previously acquired subject-specific structural whole head 1mm isotropic resolution T_1_-weighted volume for cortical unfolding (3D-MPRAGE sequence collected at 3T with 1 mm isotropic resolution, linear phase-encoding order, TE/TR=3.7/8.13 ms, FA = 8^o^, inversion time (TI)= 960 ms).

### 2.3 Data analysis

#### 2.3.1 Thermonoxious fMRI data

Data were first corrected for physiological noise using RETROICOR (Glover et al., 2000) to remove time-locked cardiac and respiratory artefacts. Distortion correction was performed using FSL's TOPUP (http://fsl.fmrib.ox.ac.uk/fsl/fslwiki/topup) using the reference SE-EPI volumes to compute the susceptibility off-resonance field (Andersson et al., 2003). Each fMRI data set was realigned to the last volume of the data set (reference EPI frame) acquired closest in time to the high-resolution T_2_*-weighted dataset and SE-EPI reference. To account for scanner drift and other low-frequency signals, all time-series were high-pass filtered (0.017 Hz cut-off) and spatially smoothed by a small kernel (Full Width Half Maximum equal to 1.5 mm). The two fMRI runs with the same stimulus condition were concatenated and converted to percent-signal change for subsequent statistical analysis. In order to identify areas exhibiting significant BOLD signal change due to thermonoxious stimulation, the concatenated data for the thenar eminence and fingertips stimuli were processed individually using the general linear model implemented in mrTools (http://www.fil.ion.ucl.ac.uk/spm/), employing a canonical double-gamma HRF and its orthogonalized temporal derivative. Resulting z-score maps were corrected for multiple comparisons comparing both false discovery rate (FDR) and family-wise error (FWE) correction. FDR-adjustment was performed using a step-up method (Benjamini et al., 2006) and FWE correction was performed across voxels using a step-down method (Holm, 1979) after estimating the number of true null hypotheses using a least-squares method (Benjamini et al., 2006; Hsueh et al., 2003). Data from the non-painful heat paradigm was analyzed in the same way to localize brain regions responding to an increase in temperature (with respect to baseline body temperature), and combined with the thermonoxious data from the same scan session to assess the contrast (Pain > Heat) and identify regions where the BOLD evoked response for pain is larger than that evoked by heat.

#### 2.3.2 Mechanoreceptive fMRI data

The fMRI time series (forward and reverse) from the vibrotactile travelling wave experiment were combined as previously described (Besle et al., 2013) to remove the effect of the haemodynamic delay in deriving the somatotopic maps. Fourier analysis was applied to obtain the phase, amplitude, and coherence to the best-fitting sinusoid at the stimulus repetition frequency. The phase indicates the temporal delay of the fMRI signal with respect to the onset of the stimulus and thus can be used to differentiate the digits. Digit somatotopic (phase) dominance maps were displayed at a coherence > 0.3.

#### 2.3.3 Projection of statistical maps onto individual flattened representations of the cortex

Cortical segmentations were obtained from the reference T1-weighted anatomical volume using FreeSurfer (http://surfer.nmr.mgh.harvard.edu/; (Dale et al., 1999). Flattened representations of the cortical regions surrounding the central sulcus and postcentral gyrus (S1) were obtained using the mrFlatMesh algorithm (VISTA software, http://white.stanford.edu/software/). In order to project the statistical maps onto flattened reconstructions of the cortical surface, statistical maps were moved from the functional EPI data space into the given individuals’ whole-head anatomical T1-weighted space in two steps: first a linear alignment matrix between the in-plane T2*-weighted anatomical volume with the T1-weighted reference volume was performed using an iterative, multi-resolution robust estimation method (Nestares and Heeger, 2000), as implemented in mrTools (http://www.cns.nyu.edu/heegerlab). Second, the reference EPI frame was non-linearly aligned to the in-plane T2*-weighted anatomical volume to account for residual distortions in the functional volume. Note that all analyses were performed in the space of the original functional data and only the resulting statistical maps were nonlinearly transformed (first into the space of the structural T2* volume, and then from the structural T2* to the subject-specific whole-head volume space) for display on the cortical surface. Visualization on the cortical surface allows to compare the location and the spatial extent of the activations evoked by thermonoxious stimulations with respect to the location of the hand-digit ROI from the vibrotactile paradigm and the subject-specific FreeSurfer labels of Brodmann areas 3a, 3b, 1 and 2 in S1, that were projected to the individual subject-specific flattened space.

## 3 Results

All subjects scanned perceived painful thermonoxious stimulation, but the degree of perceived pain was dependent on the stimulation site (see Table 1). The baseline temperature of 40 °C was not perceived as painful by any of the subjects. Temperature thresholds needed to elicit a level 7 pain rating were lower on the thenar eminence (ranging from 43.5 °C for Subject 3 to 47 °C for Subject 5), compared to the digit tips (ranging from 46 °C for Subject 3 to 48.5 °C for Subjects 1 and 5). GSR traces collected outside of the scanner at the stimulus temperature prior to the fMRI session showed an increased galvanic response a few seconds after the onset of the stimulus in all subjects and skin locations (Fig. 1.A). In two instances, the stimulus temperature was increased by 0.5 °C because the subjects reported a reduced pain sensation as compared to that outside the scanner. Due to RF interference inside the 7T scanner, the GSR recording often stopped during the actual fMRI experiment, though the subject reported the sensation as being perceived painful throughout the fMRI run. Table 1 provides details of the fMRI data collected during thermonoxious stimulation.

**Fig. 2:**
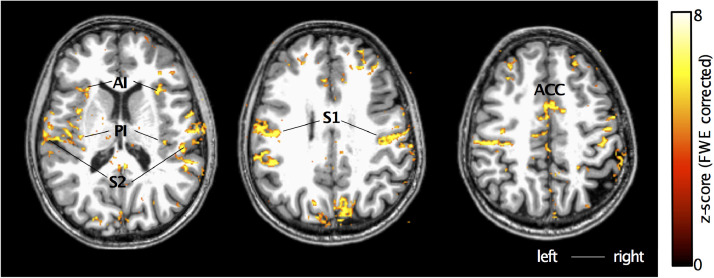
Cortical activation patterns (z-score>3.08, FWE corrected) in response to thermonoxious stimulation of the thenar eminence *of* Subject 2 overlaid onto structural T1-weighted space.

Fig. 1.A shows an example GSR trace in response to thermonoxious stimulation applied to the thenar eminence in Subject 1collected outside (top) and inside (bottom) the scanner during the fMRI run. Fig. 1.A also shows the fMRI response BOLD time series from within an ROI in the contralateral S1 (a large ROI covering both BA3a and BA3b), showing high modulation by the painful stimuli at 46 °C but no cortical response to the 42 °C non-painful stimulus, in agreement with a reduction in the GSR. Fig. 1.B shows the location of the BOLD response in horizontal, sagittal, and coronal sections. As predicted, we see prominent activity in the depth of the central sulcus contralateral to the stimulated hand (marked by red arrows). The band of thermonoxious stimulus-evoked activity is located in the posterior bank of the central sulcus and extends from its fundus all the way to the crown. The spatial extent of the activation in the contralateral S1 is shown on the cortical surface in Fig. 1.C along with the flattened representation of the central sulcus, showing activation in the posterior bank of the central sulcus (fundus indicated by the dashed line) as well as in the crown of the postcentral gyrus (indicated by the solid black line). Fig. 2 shows an example of the activation patterns evoked by thermonoxious stimulation of the thenar eminence across the brain of another subject (Subject 2). Regions modulated by painful thermal stimulation include contralateral and ipsilateral primary and secondary somatosensory cortices, as well as the posterior and anterior insula, and anterior cingulate cortex, areas known to be involved in the pain processing (Tracey and Mantyh, 2007).

**Fig. 3:**
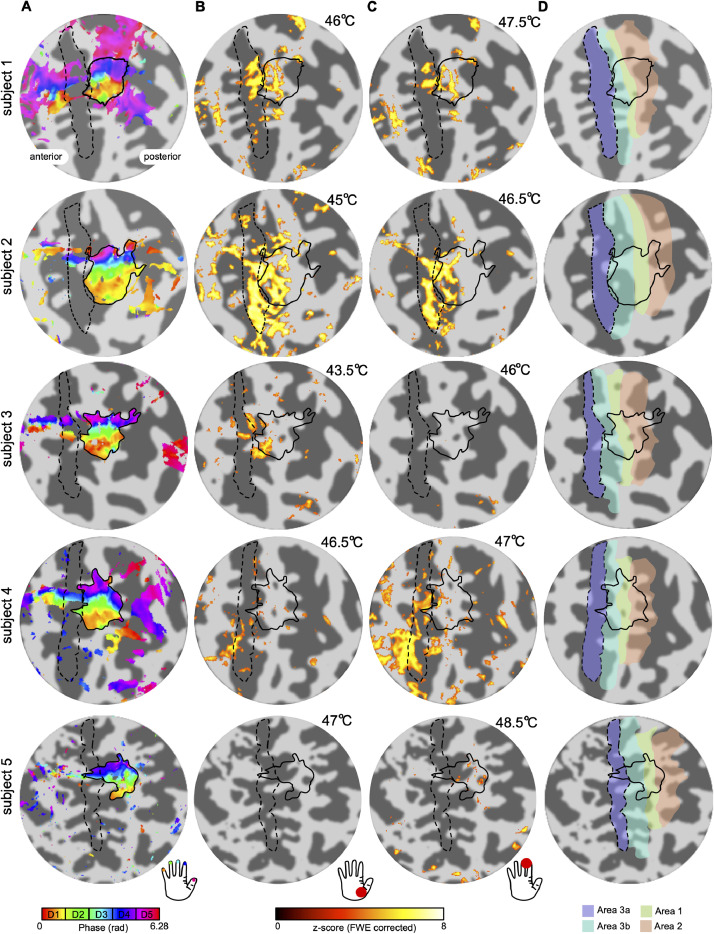
Localization of fMRI BOLD responses, obtained in all 5 subjects, to vibrotactile and thermonoxious skin stimulation and their relationship to FreeSurfer labels of Brodmann areas within the S1. (A) Somatotopic digit (phase) map (displayed at coherence value > 0.3) obtained using vibrotactile stimulation of the right hand fingertips. An orderly representation of the digits is seen in the posterior bank of the central sulcus and postcentral gyrus, corresponding to BA3b *and* BA1. The black solid outline shows the delineation of the digit territory. The black dash outline shows the delineation of BA3a as defined by the FreeSurfer label (see D). (B) Activation map for thermonoxious stimulation of the thenar eminence displayed at Z>3.08 FWE-corrected. (C) Activation map for thermonoxious stimulation of the digit tips displayed at Z>3.08 FWE-corrected. (D) FreeSurfer labels for BA3a, BA3b, BA1, and BA2.

All subjects underwent innocuous vibrotactile stimulation of the digit tips in order to directly compare the mechanoreceptive spatial localization of digit maps with nociresponsive activations. Vibrotactile stimuli were perceived as pleasant and non-painful by all subjects. Fig. 3.A shows somatotopic (phase) maps generated from the vibrotactile travelling wave paradigm for all subjects. The solid black line indicates the border of the digit somatotopic map in the posterior bank of the central sulcus and postcentral gyrus. An orderly progression of phase values encodes the location from digit 1 (yellow) to digit 5 (crimson) in the lateromedial (and inferior-to-posterior) direction across the central sulcus.

Fig. 3.B and C show the spatial extent of activations evoked in all subjects by thermonoxious stimulation of the digit tips and thenar eminence, respectively. Thermonoxious stimulation elicited strong BOLD responses in S1 of 4 out of 5 subjects in at least one of the stimulation sites (thenar eminence and digit tips). Only stimulation of the digit tip in Subject 3 and stimulation of the thenar eminence and digit tips in Subject 5 did not reach significance. Notice that for these two subjects, two stimulation sites which did not evoke significant activation within S1 were also perceived as less painful by the subject (Table 1). Furthermore, thermonoxious stimulation of the digit tips in Subject 3 failed to evoke significant BOLD responses not only in S1, but in general across the whole brain. Whilst stimulation of the thenar eminence in Subject 5 failed to evoke a significant BOLD response in S1, it did evoke significant activation in S2 and insula. In this subject, activation was weaker for thermonoxious stimulation of the thenar eminence than the digit tips, reaching significance only with a less conservative FDR-correction threshold (mean z-score of 6.6±0.4 and 6.3±0.1 (SEM) in contralateral posterior insula and S2 ROIs for digit tip stimulation compared to 5.2±0.1 and 4.9±0.3 for thenar eminence stimulation). The thenar eminence stimulation also produced clear responses in the GSR trace.

Fig. 3.B and C show that in all the fMRI scanning sessions in which thermonoxious skin stimulation evoked a significant BOLD response in the contralateral S1, this response was dominated by responses deep in the posterior bank of the central sulcus, as predicted. Only a small fraction of the response was located superficially in the central sulcus or on the crown, overlapping digit maps defined by the vibrotactile travelling wave. The thenar eminence and fingertip responses were similar in their S1 locations, differing primarily in the significance of the BOLD response. When comparing the spatial extent of thermonoxious evoked activity with FreeSurfer labels for Brodmann areas in S1 (Fig. 3.D, as well as dash outlines in Fig. 3.B and C), it can be seen that the thermonoxious response largely overlaps with putative BA3a, whilst vibrotactile based digit maps largely overlap with putative BA3b, BA1 and BA2, and not BA3a.

**Fig. 4:**
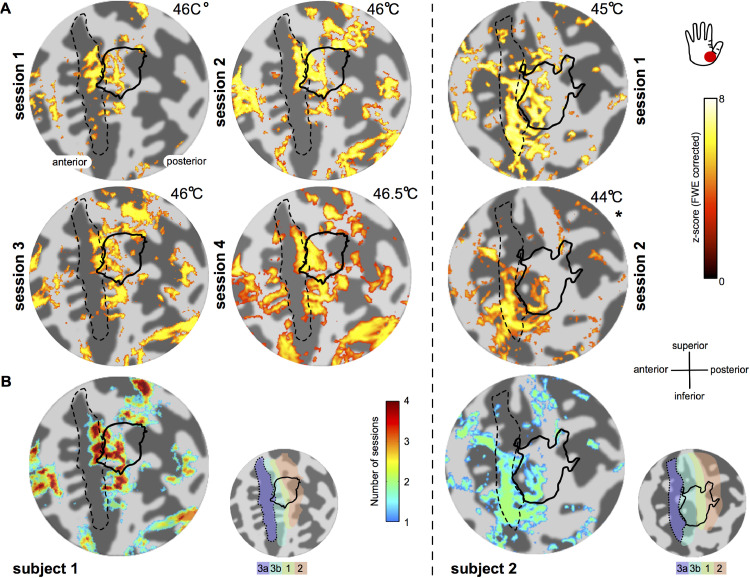
Reproducibility of S1 responses to thermonoxious stimulation across multiple scanning sessions. (A) Statistical *z*-score activation maps (displayed at *z*>3.08 FWE corrected) in response to thermonoxious stimulation of the thenar eminence are shown on flattened representation of the central sulcus for subject 1 (left) and subject 2 (right.) (*) Displayed at *z*>3.08 FDR-corrected. The number in the top right corner of each sub panel indicates the stimulation temperature. The black dashed outline indicates putative BA3a based on FreeSurfer probabilistic map. The black solid line indicates the border of the vibrotactile digit map. (B) Overlap of stimulus-activated areas across *n*=4 scanning sessions for Subject 1 and *n*=2 scanning sessions for Subject 2. Conjunction map was formed based on responses with a significant threshold *z*>3.08 after family-wise correction (Subject 1) and false-discovery correction (Subject 2).

The fMRI responses elicited by thermonoxious skin stimulation were reproducible. Fig. 4 shows the similarity of activation maps in contralateral S1 collected across multiple scanning sessions in two subjects. Note the lower significance for the second session of Subject 2 (Fig. 4.A), where the stimulation was performed at a lower temperature. Despite this, the spatial extent of the activation within the central sulcus is similar across the two sessions. Fig. 4.B shows large overlap of activation across scanning sessions for both subjects.

**Fig. 5:**
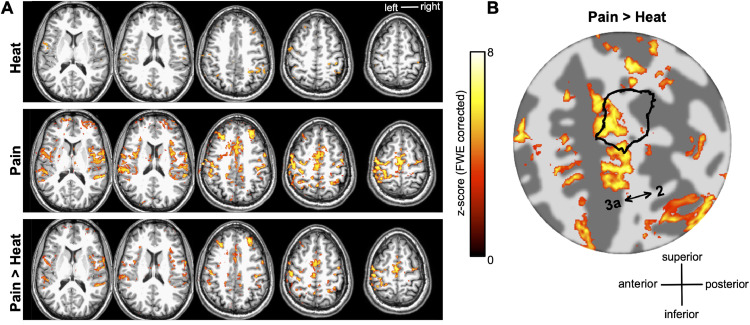
(A) Cortical activation in response to innocuous thermal (hot) stimulation (Heat, top row) of the thenar eminence compared to thermonoxious stimulation (Pain, middle raw) on the same skin surface (subject 1) overlaid onto structural T1-weighted space. The contrast Pain>Heat (bottom row) showing areas where thermonoxious BOLD evoked responses are larger than BOLD responses induced by a change in temperature is very similar to the Pain contrast (both displayed at *z*>3.08 after FWE correction, while the Heat contrast map is uncorrected). (B) Statistical map for contrast Pain>Heat overlaid onto a flattened representation of the central sulcus. Notice that the activation extends anteriorly and inferiorly with respect to delineation of the tactile digit territory (shown by the black solid outline).

Fig. 5 compares the cortical response to innocuous thermal stimulation (increasing temperature by 5 °C from 37 °C to 42 °C) of the thenar eminence to thermonoxious stimulation (increasing temperature by 6.5 °C from 40 °C to 46.5 °C) applied to the same skin site (Subject 1). The contrast Pain>Heat shows areas where thermonoxious BOLD evoked responses are larger than those induced by a change in temperature, this yields a very similar map to the Pain contrast alone.

## 4 Discussion

Here we have used high resolution 7T fMRI to study the cortical responses to thermonoxious stimulation compared to innocuous mechanical stimulation. Thermonoxious stimulation engaged the pain network within S2, posterior insula, and anterior cingulate, in agreement with primate studies (Biedenbach et al., 1979; Chudler et al., 1990; Dong et al., 1989; Kenshalo et al., 1988; Kenshalo and Isensee, 1983; Robinson and Burton, 1980) and fMRI studies in humans (Apkarian et al., 2005; Geuter et al., 2020). Importantly, nociceptive responses were also elicited in S1, in agreement with previous fMRI studies that employed comporable thermonoxious stimuli (Becerra et al., 2001; Chen et al., 2002; Downar et al., 2003; Fairhurst et al., 2012; Gelnar et al., 1999; Moulton et al., 2012; Tseng et al., 2013; Upadhyay et al., 2010).

### 4.1 Localization and comparison of vibrotactile and thermonoxious responses in S1

The two paradigms used in this study, thermonoxious and non-noxious mechanical skin stimulation, engage different submodalities of somatosensory afferents, which target distinct cortical areas. Mechanical stimulation of the digits, here delivered in a form of vibrotactile stimulation, has been shown to yield robust activation in BA3b and BA1 at 7T (Besle et al., 2014; Kolasinski et al., 2016; Martuzzi et al., 2014; Puckett et al., 2017; Stringer et al., 2011) and BA2 to a lesser extent (Martuzzi et al., 2014; van der Zwaag et al., 2015). In agreement, in our study the delineation of the hand area obtained by vibrotactile stimulation of the digit tips (tactile hand region) lies predominantly within putative BA3b and BA1 according to the FreeSurfer probabilistic labels (Fig. 3.A) and partially extends into putative BA2, but it has little overlap with BA3a. It should be noticed that the travelling-wave paradigm results in this mapping being insensitive to the non-specific BOLD contributions from large veins that drain blood from across the whole hand representation in S1, thus suppressing the venous signal modulations. It is possible that the thermonoxious stimulation block design does give rise to some venous signals, but we aimed to reduce such possible effects by using a baseline temperature of 40 °C rather than neutral temperature of 32 °C.

Since the goal of this study was to test the hypothesis—posited by non-human primate studies (Vierck et al., 2013; Whitsel et al., 2019)—that the human central sulcus contains a slow-responding C-dominated nociresponsive region near its fundus in BA3a, our thermonoxious stimuli were optimized to evoke a prominent response in this region. Knowledge that in monkeys this region exhibits prominent temporal summation in its response to thermonoxious stimuli and does not respond effectively to brief stimuli, meant it was most important for our painful heat stimuli to be sufficiently long (such as 5 s, using monkey studies as a guidance). Our ramping heat stimuli activate not only C-nociceptors but also Aδ nociceptors and can be expected also to evoke prominent response in the fast-responding Aδ-dominated nociresponsive region in BA3b-BA1, higher up in the posterior bank of the central sulcus and on the crown of the postcentral gyrus. Indeed, our results show that BOLD responses evoked by the thermonoxious stimulation were distributed throughout the depth of the central sulcus, overlapping primarily with FreeSurfer-demarcated BA3a and BA3b. It should be noted that labels of the anatomical Brodmann areas (3a, 3b, 1 and 2) provided by FreeSurfer are probabilistic based on ex-vivo histological measurements (Amunts et al., 2007; Geyer et al., 1999, 2000; Grefkes et al., 2001) and not ground truth subject-specific anatomical areas, hence they need to be interpreted with caution, particularly given that the size and position of the anterior and posterior borders of area 3a relative to the fundus of the central sulcus have been shown to vary greatly across individuals (Geyer et al., 1999, 2000).

BOLD responses evoked by the thermonoxious stimulation partially overlap with the tactile hand area derived from the BOLD responses evoked by vibrotactile stimulation (Fig. 3.B and C). Activation to noxious stimulation within the tactile hand is limited mostly to its anterior part, spanning predominantly FreeSurfer-demarcated BA3b; it then extends anteriorly towards the depth of the central sulcus in putative BA3a with the responses in some subjects also extending more laterally (Subjects 1, 2 and 4 (Figs. 3 and 4)). These findings of a partial overlap of nociceptive and tactile regions in human S1 agree with non-human primate studies. Nociresponsive neurons in the BA3b-BA1 region, predominantly of the wide dynamic range (WDR) type, are embedded among Aβ mechanoreceptor-innervated neurons, which are highly responsive to vibrotactile stimuli as used in our study (Chudler et al., 1990; Kenshalo and Willis, 1991; Kenshalo et al., 2000). Thus, the Aδ-dominated BA3b-BA1 nociceptive region will respond to both thermonoxious and vibrotactile stimuli, as observed in this study. In contrast, neurons in the C-dominated nociresponsive region show little responsivity to vibrotactile stimulation (Tommerdahl et al., 1996, 1998; Whitsel et al., 2009), as observed in our study with thermonoxious stimulation recruiting cortical regions anterior to the tactile hand area.

Thermonoxious stimulation of both thenar eminence and digit tips evoked robust activation maps in Subjects 1, 2 and 4. These were similar in spatial extent but differed in statistical significance, and this may reflect the underlying differences in the intensity of the perceived pain. The finding that the thenar eminence and the stimulated tips of digits 2 and 3 span similar cortical territories is not surprising considering that they occupy nearby locations in the S1 somatotopic map (McKenna et al., 1982).

### 4.2 Comparison of noxious S1 activation to previous studies

Early fMRI studies on cortical pain processing have reported conflicting results on the involvement of S1 in pain processing, with some studies failing to record any nociceptive activity in S1 (see critical reviews, addressing possible reasons for such failures, by Apkarian et al., 2005; Bushnell et al., 1999; Peyron et al., 2000). Reasons suggested to explain such variable outcomes include lability of S1 responses due to attentional and cognitive factors; inter-subject variability in the response location and limited acquired spatial resolution preventing relatively small nociceptive maps to be revealed in traditional group averaging in fMRI studies (Mancini et al., 2012); anatomical variability in the extension of the postcentral gyrus limiting the detectability of activity in S1 using MEG, with Kanda et al. (2000) detecting S1 activity in 7/12 subjects; and Ploner et al. (2002) argued that the short duration of S1 activation related to 1st/sharp pain is less likely to be detected by fMRI than longer-lasting activation of S2 and ACC based on MEG data. In the present fMRI study, significant BOLD responses were observed in the FreeSurfer-demarcated BA3a in the depth of the central sulcus in 13 out of 16 (80%) thermonoxious stimulation sessions in 4 out of 5 subjects, and were reproducible across sessions performed months apart in the same subject. Although it might not necessarily be related, in 2 of the 3 failed sessions the subjects reported that the stimuli were “not very painful” (Table 1).

Cheng et al. (2015) identified a factor that might underlie, in part, the inter-subject variability of S1 responses to noxious stimulation: i.e., their propensity for temporal summation of pain. Individuals vary greatly on the antinociception-pronociception spectrum, in particular how readily their experienced pain becomes magnified with repeated or continuing noxious stimulation (Yarnitsky et al., 2014). In their combined psychometric/fMRI study of healthy individuals, Cheng et al. (2015) showed that individuals’ propensity for temporal summation of pain was positively correlated with the strength of their resting-state functional connectivity between the thalamus and BA3a, but not BA3b or BA1. Knowing that BA3a response to noxious stimulation relies greatly on slow temporal summation (Vierck et al., 2013; Whitsel et al., 2009), it is tempting to speculate that a failure to detect BOLD response to noxious stimulation in BA3a (such as, for example, Subject 5) might be a sign that the tested individual comes from the anti- side of the antinociception-pronociception spectrum.

Neuroimaging studies comparing the location of responses in S1 for noxious and innocuous tactile stimulation of the hand have been inconsistent in their findings. Some studies reported the nociceptive region to be more medial than the tactile region (Coghill et al., 1994; Kanda et al., 2000; Ploner et al., 2000), whereas others report no difference between the nociceptive and tactile regions (Chen et al., 2002; Inui et al., 2003; Mancini et al., 2012). In our study, we find that nociceptive and tactile regions overlap partially, with a major component of the nociceptive region lying either directly anterior to the tactile region in some individuals or anterior and lateral in the others.

The involvement of S1 in pain processing associated with unmyelinated C-fibres has been investigated in a number of studies. Functional studies of C-fibre-related cortical responses have demonstrated activation of S1 using PET (Andersson et al., 1997; Iadarola et al., 1998; Petrovic et al., 2000), SPET (Di Piero et al., 1994) and MEG (Ploner et al., 2002; Tran et al., 2002). However, the spatial resolution of those imaging studies was too coarse to infer which part of S1 was the focus of the activity. An EEG study of Jin et al. (2018) saw peaks of Aδ- and C-specific responses in S1, but noxious stimuli were in the form of single 4ms duration laser pulses, which are not likely to evoke a noticeable response in BA3a. fMRI studies targeting C-nociceptors with long-duration noxious heat stimuli have also reported activation in S1, either finding nociceptive responses on the crown but not in the depth of the central sulcus (Gelnar et al., 1999), or reporting the location to be highly varied across subjects (Chen et al., 2002), or not specifying the location within S1 (Becerra et al., 2001; Downar et al., 2003; Fairhurst et al., 2012; Helmchen et al., 2006; Moulton et al., 2012; Nahman-Averbuch et al., 2014; Tseng et al., 2013; Upadhyay et al., 2010). These studies describe an initial ON-peak in the BOLD response around 6 s after the stimulus onset, followed by a dip and then a slow second rise, and termination with an OFF-peak around 6s after the stimulus end. The slow second rise is consistent with the expected slow buildup of activity in the nociresponsive BA3a region.

### 4.3 C-dominated nociresponsive region at S1-M1 border: a separate cortical area?

Although it is nominally assigned to BA3a, the nociresponsive region at the S1-M1 border occupies a transitional zone with a distinctive mix of cytoarchitectonic features (Whitsel et al., 2019). Its nociceptive afferent input comes from lamina I of the spinal cord dorsal horn (Craig, 1995, 2014; Dum et al., 2009), which in turn receives its main peripheral input from small-diameter afferents associated with the sensations of cooling, warmth, itch, affective touch, muscle ache, fatigue, skin and internal organ pain (Craig, 2003, 2015; Craig et al., 2001; Craig and Andrew, 2002; Craig and Blomqvist, 2002). Neurons in this region are minimally affected by non-noxious tactile or proprioceptive stimuli (Whitsel et al., 2009). Thus it appears that BA3a in primates contains two separate areas: (1) an anterior “interoceptive” area, which is dedicated to spinal lamina I C-afferent inputs ascending the spinothalamic tract; and (2) a posterior “proprioceptive” area, which is dedicated to Aβ proprioceptive inputs ascending in the dorsal columns. Given its distinct combination of cytoarchitecture, afferent connectivity, and functional properties, and to distinguish it from the proprioceptive BA3a (Krubitzer et al., 2004), it might be appropriate to recognize the anterior part of BA3a at the S1-M1 border as a separate cortical area – area 3c, or BA3c (Jones and Porter, 1980).

### 4.4 Functional role of BA3c

Kleist (1934) was the first to hypothesize, based on his observations of the permanent loss of pain sensibility in some World War I soldiers following local brain injuries extending deep into the posterior bank of the central sulcus, that pain sensations might be localized in a BA3a subregion. Similar observations were later made by Russel (1945) in World War II soldiers, suggesting that functional integrity of the cortex in the depth of the central sulcus might be necessary for an individual to be able to perceive pain (Perl, 1984; Whitsel et al., 2019).

This suggestion is supported by studies performed in rats, in which the dysgranular transition from agranular M1 to granular S1 is recognized as a separate region called the *transitional zone*, TZ (Chapin and Lin, 1984). This region is a rat equivalent of primates’ slow C-dominated nociresponsive BA3c: rat TZ neurons exhibit all the same functional properties as neurons in primate BA3c (Favorov et al., 2019). Pointing to its importance for nociception and nocifensive behavior, selective inactivation of rat TZ by cooling or lidocaine injection suppresses the nociceptive flexor withdrawal reflex, indicating that TZ exerts a tonic facilitatory influence over spinal cord neurons producing the reflex (Favorov et al., 2019). Inactivation of TZ was found to produce a loss of responsivity of nociceptive neurons in the contralateral dorsal horn to thermonoxious stimulation of their receptive fields, raising a possibility that TZ in rats (and by extension, BA3c in primates) acts as a cortical positive feedback “pain booster” amplifying noxious inputs reaching the dorsal horn from C-nociceptor afferents in the body (Whitsel et al., 2009, 2019).

A different but complementary mechanism has been identified by Singh et al. (2020), who showed that rat anterior cingulate cortex (ACC) receives direct excitatory synaptic connections from S1. These S1-ACC projections not only increase the response of ACC neurons to noxious stimulation but also enhance aversive behavioral responses to pain. Conversely, optogenetic inhibition of the S1-ACC projections effectively relieves the aversive component of acute and, clinically more important, chronic pain. It remains to be determined whether the relevant S1 connections come from granular S1 (homolog of primate BA3b/1) or TZ.

While we focused on nociceptive properties of BA3c in this paper, it is quite possible that this area might include populations of neurons with other interoceptive properties, reflecting not only nociceptors but also other C-afferent submodality classes of spinal lamina I neurons, and thus associated with the sensations of cooling, warmth, itch, affective touch, muscle ache, fatigue, etc. None of these submodalities have been explored in BA3c yet, but it is important to do so because, if found, this would broaden our view of BA3c as engaged in interoception and control of autonomic nervous system.

### 4.5 Clinical significance of BA3c

In the mid-20th century, selective surgical ablations of the S1 cortex were used as a therapeutic means of last resort in treating patients suffering from severe chronic pain, most commonly the phantom limb pain. This procedure, called *postcentral topectomy*, was successful in curing some but not all patients and it was eventually abandoned. In light of the discovery of nociresponsive BA3c, Challener and Favorov (2020) reviewed every postcentral topectomy case available in the neurosurgical literature. They found 17 full-text reports from 16 different surgical teams describing outcomes of the procedure in 27 patients. Among those, in 5 patients (19%) the procedure either failed to abolish the targeted chronic pain or the pain returned to its preoperational levels. In the other 22 patients, their pain stayed abolished or at least significantly reduced as of the last evaluation (which was one year or more for 9 patients). The varied outcomes of the postcentral topectomy might be due to whether the ablation included BA3c (Vierck et al., 2013; Whitsel et al., 2019): when ablation extended deep into the central sulcus and included BA3c, the chronic pain loss was permanent, whereas when an ablation of the postcentral gyrus was too shallow, the loss of pain was, at most, transient until BA3c recovered from indirectly induced trauma. If this explanation for the unsatisfactory outcomes of the postcentral topectomy is correct, it should be possible to greatly improve the success rate by using high-resolution fMRI to localize BA3c. Such a targeted inactivation of BA3c might make this procedure a highly attractive means of treating some of the otherwise intractable chronic pain conditions, especially in combination with transcranial MR-guided focused ultrasound (Challener and Favorov, 2020).

## 5 Conclusion

This study was conducted to investigate whether humans have a cortical region in the depth of the central sulcus that is engaged in nociception. The existence of such a region has been predicted by the presence of a C-nociceptor innervated region at the S1-M1 border in rat and monkey primary somatosensory cortex. Thermonoxious skin stimulation evoked 7T BOLD responses in the depth of the posterior bank of the central sulcus in humans, in non-overlapping regions compared with somatotopic maps generated using innocuous vibrotactile stimulation of the digits. A precisely targeted inactivation of this region, visualized with the use of high-resolution thermonoxious fMRI, might offer effective means of treating some pathological pain conditions.

## CRediT authorship contribution statement

**Rosa M. Sanchez Panchuelo:** Data curation, Formal analysis, Methodology, Conceptualization, Funding acquisition, Writing - original draft. **Sally Eldeghaidy:** Data curation, Formal analysis, Methodology, Writing - review & editing. **Andrew Marshall:** Conceptualization, Writing - review & editing. **Francis McGlone:** Conceptualization, Writing - review & editing. **Susan T. Francis:** Data curation, Methodology, Conceptualization, Resources, Writing - review & editing. **Oleg Favorov:** Conceptualization, Methodology, Writing - original draft.

## Declaration of Competing Interest

None.
